# Recombinant fragment of human surfactant protein D to prevent neonatal chronic lung disease (RESPONSE): a protocol for a phase I safety trial in a tertiary neonatal unit

**DOI:** 10.1136/bmjopen-2024-086394

**Published:** 2024-08-16

**Authors:** Reena Bhatt, Jens Madsen, Tania Castillo-Hernandez, Kathy Chant, Hakim-Moulay Dehbi, Neil Marlow, Howard Clark

**Affiliations:** 1 Neonatal Intensive Care Unit, University College London, London, UK; 2 Department of Neonatology, Institute for Women's Health, University College London, London, UK; 3 Comprehensive Clinical Trials Unit, University College London, London, UK

**Keywords:** immunology, neonatal intensive & critical care, respiratory medicine (see thoracic medicine)

## Abstract

**Introduction:**

Chronic respiratory morbidity from bronchopulmonary dysplasia (BPD) remains the most common complication of preterm birth and has consequences for later respiratory, cardiovascular and neurodevelopmental outcomes. The early phases of respiratory illness are characterised by rapid consumption of endogenous surfactant and slow replenishment. Exogenous surfactant is routinely administered to infants born before 28 weeks of gestation as prophylaxis. Endogenous surfactant includes four proteins, known as surfactant proteins (SPs) A, B, C and D. Current bovine-derived and porcine-derived surfactant preparations only contain SPs B and C. SP-D has a key role in lung immune homeostasis as part of the innate immune system. Laboratory studies using recombinant SP-D have demonstrated reduced inflammation, which may be a pathway to reducing the associated morbidity from BPD. RESPONSE uses a recombinant fragment of human SP D (rfhSP-D), in a phase I safety and dose-escalation trial as the first stage in determining its effect in humans.

**Methods and analysis:**

This is a single-centre, dose-escalation, phase I safety study aiming to recruit 24 infants born before 30 weeks gestation with respiratory distress syndrome. In addition to routine surfactant replacement therapy, participants will receive three doses of rfhSP-D via endotracheal route at either 1 mg/kg, 2 mg/kg or 4 mg/kg. The study uses a Bayesian continual reassessment method to make dose escalation decisions. Dose-limiting events (DLE) in this trial will be graded according to the published Neonatal Adverse Event Severity Score. The primary outcome of this study is to evaluate the safety profile of rfhSP-D across each dose level based on the profile of DLE to establish the recommended phase 2 dose (RP2D) of rfhSP-D.

**Ethics and dissemination:**

The RESPONSE study has received ethical approval from London-Brent NHS Research Health Authority ethics committee. Results from the study will be published in peer-reviewed journals and presented at national and international conferences.

**Trial registration numbers:**

ISRCTN17083028, NCT05898633.

**Protocol version:**

RESPONSE Protocol V.4.0 24th July 2024.

**STRENGTHS AND LIMITATIONS OF THIS STUDY**

- This study uses the International Neonatal Consortium Neonatal Adverse Event Severity Score (NAESS) which is specific to this population and allows a better grading and understanding of the adverse events and progression of the trial. This scoring system unlike others, for example, Common Terminology Criteria for Adverse Events takes into account age-appropriate behaviour, such as, feeding and physiological parameters. Although the NAESS has not been rigorously validated, it is well placed to improve the quality of drug evaluation in this highly vulnerable population.
- This is a safety study aiming to establish a recommended phase II dose of a novel therapy in a highly vulnerable population affected by Bronchopulmonary Dysplasia which has a significant impact on long-term lung health.
- This study uses Bayesian analysis which uses prior cohort data to inform the ongoing dose escalation.
- This is a single-centre study which may affect recruitment and the population characteristics.

## Introduction

### Clinical need for study

 The introduction of exogenous surfactant replacement therapy has significantly improved mortality in extremely preterm infants, those born before 28 weeks of gestation.^1^ Despite this, chronic respiratory morbidity from bronchopulmonary dysplasia (BPD) remains the most common complication of very preterm birth. BPD may be formally defined by the persisting need for respiratory support past 36 weeks postmenstrual age (PMA).^2^ It affects up to 75% of extremely preterm infants,^3^ with decreasing prevalence with increasing gestational age (GA).^4^ The pathogenesis of BPD is complex and multifactorial, involving lung immaturity, infection, inflammation, oxygen toxicity and ventilator-induced injury.

Unlike when first described, BPD is now rarely seen in infants born at more than 1200 g or after 30 weeks of gestation^2 5 6^ due to the introduction of antenatal steroid administration, surfactant replacement therapy, improved ventilation strategies and better nutrition.^7 8^ The prevalence of BPD has not fallen as expected.^8^,^10^ It can be argued that with advances in neonatal care leading to increased survival of infants at greatest risk of BPD, the prevalence may increase in years to come presenting a challenge for healthcare systems worldwide. Furthermore, BPD has lifelong consequences, with respiratory impairment that has important implications for adult clinicians, tracking through to adult life^11 12^ and neonatal BPD is also a marker for adult cognitive, educational and behavioural impairment with implications for health, wealth and relationships for life.^13^

As the mean GA of neonatal populations has fallen with increasing survival, the pathophysiology of chronic respiratory disease in very preterm populations has changed. Whereas the original descriptions of BPD related the occurrence and progression primarily to barotrauma from mechanical positive pressure ventilation,^14^ with increasing immaturity the profile of causation has changed, and this ‘new’ BPD^15^ is primarily found among extremely preterm infants. The primary driver in its development is lung inflammation, subject to the other risks referred to above. The disease is characterised by developmental arrest of lung tissue and a loss of alveolar septation by impairing alveolar crest development. This interruption in normal lung development with superimposed inflammation, oxygen toxicity and pressure-induced changes (barotrauma, volutrauma, atelectotrauma) completes the clinical picture.

Postnatal pulmonary inflammation is due to an imbalance in humoral factors favouring a proinflammatory response^16 17^ and increased presence of inflammatory cells in the airway.^18^ Inflammation, secondary to positive pressure ventilation, oxygen therapy or infection, may have a further impact on the cytokine profile and the interruption of lung development. The overwhelming evidence for inflammation as a causal mechanism in the development of BPD suggests that early anti-inflammatory therapies might reduce the frequency and severity of the condition. Identification of potential therapeutic targets remains a goal to reduce the frequency of BPD in high-risk infants. Naturally occurring surfactant proteins D (SP-D) has gained increasing interest as a potential immunotherapy to dampen the proinflammatory cascade and facilitate lung repair, thus reducing the frequency and severity of lung disease. In turn, this may have important long-term benefits for the child.

### Surfactant protein D

Mammalian surfactant comprises largely phospholipids (80%), neutral lipids (10%) and SPs (10%), dipalmitoylphosphatidylcholine being the primary surface-active component at the alveolar surface.^19^ Four SPs are found in surfactant, SP-A, SP-B, SP-C and SP-D. SP-B and SP-C are hydrophobic and their role is largely to stabilise the lipid monolayer formed at the air-liquid interface by stimulating phospholipid adsorption and reducing surface tension. Due to their hydrophobic nature, these SPs are easily extracted from bovine or porcine sources and present in widely used commercial surfactants. In contrast, SP-A and SP-D are hydrophilic and are not present in the surfactant preparations currently used in clinical practice.

SP-D is an essential lung component and functions to keep the lungs in a hyporesponsive state at rest, free from aberrant inflammation and infection. The actions of SP-D include aggregation of pathogens, antimicrobial activity against pathogens such as *Klebsiella,* increased phagocytosis and clearance of apoptotic cells and regulation of mediator production.^20^ SP-D consists of four main regions which include an N-terminal domain, a collagenous tail, a neck region and a carbohydrate recognition domain (CRD); it exists as a trimer. Through its carbohydrate recognition domain, SP-D binds carbohydrates in a calcium-dependent manner^20 21^ and via the N-terminal region, the trimeric units oligomerise to give rise to a dodecameric cross-like structure. These can further form oligomers or ‘stellate multimers’, which increase the strength to bind carbohydrates and agglutinate various pathogens.^20^

### SP-D levels in preterm infants and evidence for recombinant fragment SP-D as a therapeutic agent

Bronchoalveolar lavage (BAL) samples taken from preterm infants over the first few days after birth have demonstrated low concentrations of SP-D in association with respiratory distress syndrome (RDS) that were associated with an increased risk of BPD.^22 23^ Binding assay studies evaluating the lectin activity of SP-D demonstrate that the SP-D present in the BAL of preterm infants was less effective than that in term infants.^23^ Sepsis in preterm infants can be life-threatening and contributes significantly to the inflammation seen in BPD. Further, SP-D concentrations increase in preterm infants in the presence of sepsis, demonstrating its potential role as an acute phase reactant.^24^ Given the known interactions of SP-D with bacterial, viral and fungal pathogens,^20 25^ intervention with SP-D would be expected to promote their clearance in this vulnerable population and reduce further damage. Finally, in SP-D knock-out mouse models,^26^ emphysematous changes are seen that are similar to those seen in the lungs of preterm infants.

Given these homeostatic and anti-inflammatory roles of SP-D, it is an attractive target for therapy, and if administered early to preterm infants there would be a reduction in inflammation by downregulation of the proinflammatory signalling pathways in addition to interaction with common pathogens that induce inflammation such as *Escherichia coli.* In vivo studies in preterm lambs given recombinant full-length SP-D in addition to commercially available surfactant showed a clear reduction in the proinflammatory cytokines such as interleukin-8 (IL-8),^27^ which provides encouraging data for its potential clinical use in this population.

In practice, the properties of full-length SP-D (including varying degrees of oligomerisation, limited solubilisation and potential aggregation at higher concentrations) make it difficult to develop a stable preparation that could be administered. Therefore, recombinant fragments of human SP-D have been explored in translational models as a potential therapy for BPD. Preclinical data showed the efficacy of rfhSP-D treatment in reducing and correcting inflammation in chronic inflammatory lung disease caused by SP-D deficiency. SP-D knock-out mice develop symptoms of chronic obstructive pulmonary disease and emphysema relevant to BPD, which are correctable following treatment with recombinant SP-D.^26 28^

A stable form of rfhSP-D has been produced using a mammalian cell line and purified using affinity chromatography using a N-Acetylmannosamine (ManNAc)-coupled matrix as described previously.^29^ The recombinant fragment comprises the neck, CRD and eight gly-Xaa-Yaa repeats similar to that described for a bacterially expressed recombinant fragment of human SP-D.^30^ The CRD is the functional anti-inflammatory and anti-infective part of the protein without the long collagenous tail and the suggested proinflammatory N-terminal region.^30^ The rfhSP-D proposed as an investigational medicinal product (IMP) retains its anti-inflammatory properties when used as an adjunct to exogenous surfactant therapy administered via an endotracheal tube in a well-established translational model using preterm ventilated lambs.^31^ The endotoxin content is less than 0.05 EU/mg rfhSP-D.

### Justification for the dosage regimen in the safety trial

The proposed regimen is based on the estimation of human equivalent dosages based on effective dosing in animals. In murine studies, the replacement dose of rfhSP-D was 10 µg daily. Assuming an average mouse weight of approximately 10–20 g, this approximates to 1–2 mg/kg per day. The effective dose of rfhSP-D in the preterm lamb has been estimated to be 1.5 mg/kg (unpublished data). In current practice, the administration of 100–200 mg/kg of surfactant replacement would contain 1–4 mg/kg if a naturally occurring product was used. Hence, after due consideration, we elected to trial three potential dose levels of rfhSP-D, namely 1, 2 and 4 mg/kg/dose.

### Study objectives

RESPONSE is a phase I study and aims to assess the safety of 3 intratracheal dose levels (1 mg/kg/dose, 2 mg/kg/dose and 4 mg/kg/dose) of rfhSP-D in preterm ventilated infants (23 weeks - 29 weeks and 6 days) at risk of BPD.

The primary objectives are as follows:

- To assess the safety profile of rfhSP-D across three dose levels based on the occurrence of dose-limiting events (DLEs) as defined below.
- To establish the recommended phase 2 dose (RP2D) of rfhSP-D for preterm infants born before 30 weeks of gestation.

Secondary objectives are as follows:

- To evaluate systemic absorption of rfhSP-D using serial measurements of SP-D in plasma and its continued presence in tracheal fluid.
- To determine the effect of rfhSP-D on inflammatory markers in lung secretions and plasma (eg, cell counts of neutrophils, macrophages, lymphocytes and cytokine; IL-8, IL-6, IL- 1).
- To compare the clinical effects of intratracheal administration of rfhSP-D on physiological and intensive care parameters in treated infants in this trial with non-treated infants from a parallel observational cohort study of untreated infants.

## Methods and analysis

### Trial design

**Figure 1:**
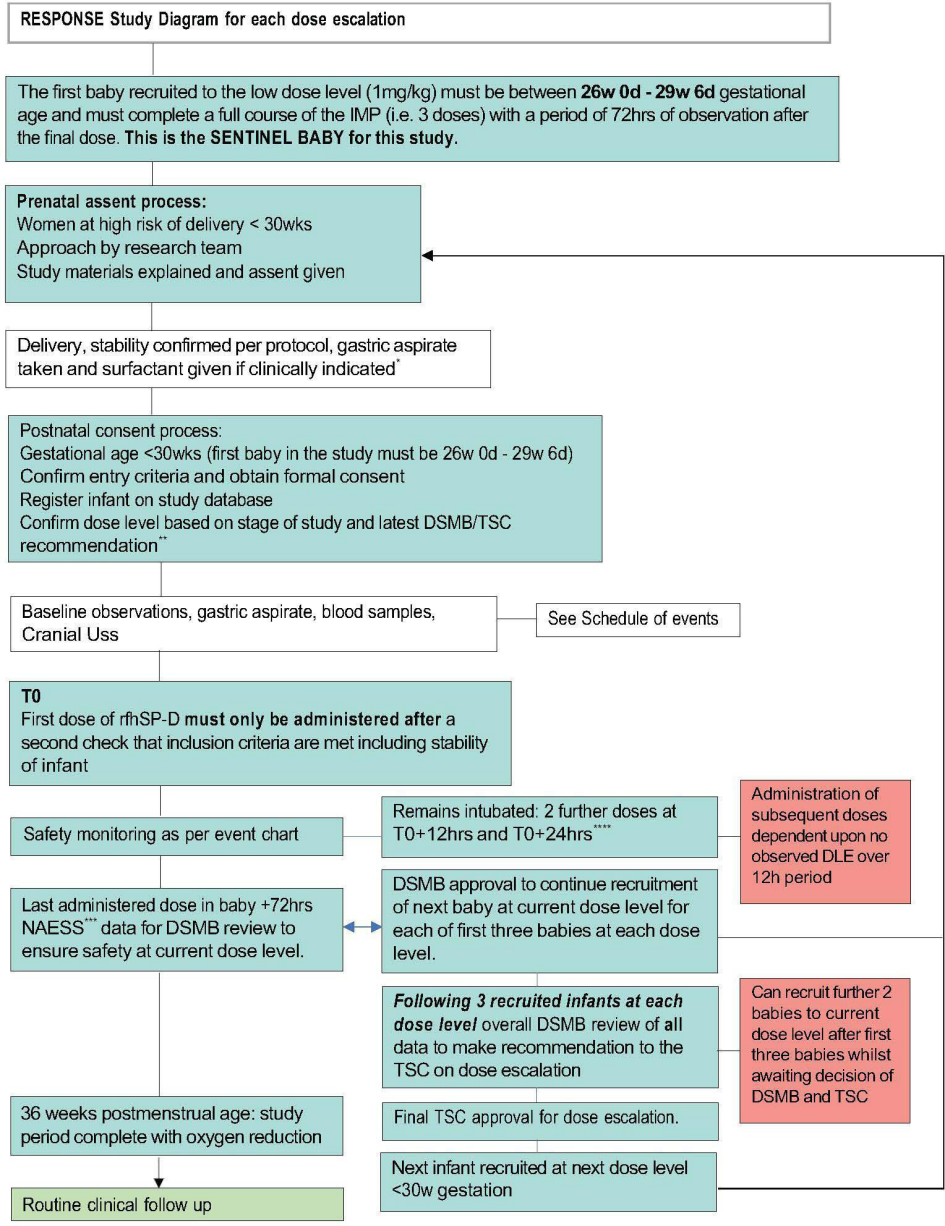
Dose escalation process in RESPONSE using rfhSP-D in preterm infants at risk of BPD. *Via Endotracheal tube only. **First iteration will be at 1mg/kg/dose, subsequent iterations 2 mg/kg/dose and 4 mg/Kg/dose. ***Neonatal Adverse Event Severity Score (NAESS). ****administration window of +2 hours. BPD, bronchopulmonary dysplasia; DLE, dose-limiting event; DSMB, data and safety monitoring board; TSC, trial steering committee.

The study will be conducted in a single-centre, tertiary level 3 neonatal intensive care unit. The study was opened on 6 February 2024 and has a proposed 12-month recruitment period. We aim to recruit 24 infants born between 23 weeks+0 days and 29 weeks+6 days gestation. To date, four infants have been recruited to the study. This study uses a Bayesian continual reassessment model (CRM),^32^,^34^ a model-based design that informs how the dosage of rfhSP-D should be adapted for the next participant cohort based on past trial data. For this first-in-human study, a dose escalation design will be used (figure 1).

The three dose levels to be considered are 1 mg/kg/dose, 2 mg/kg/dose and 4 mg/kg/dose. Participants will be enrolled at each dose level with a minimum of three participants per dose level. Each participant will receive three doses of rfhSP-D at 0 hours, 12 hours and 24 hours provided that they continue to meet the inclusion criteria and are clinically stable. The first dose of rfhSP-D should be administered after standard surfactant therapy has been given. Whether or not the dose level is escalated will depend on the occurrence of DLEs in all current participants and the doses they have received. A model will be used to estimate the risk of DLE per dose level. Initial estimates of these risks will be updated using data collected throughout the trial. A one-parameter empiric model will be used to describe the relationship between the dose and the probability of observing a DLE. The CRM model will not allow dose-skipping. The target level of DLEs level is set at no greater than 20%. Before the trial, the parameter of the model will be assigned a non-informative prior distribution and initial estimates of DLE probabilities will be derived using model calibration. The recommended phase 2 dose will be defined by considering safety and will be the highest dose level that has an estimated probability of DLE closest but below the target DLE level of no greater than 20%.

#### Dose escalation procedure

**Figure 2:**
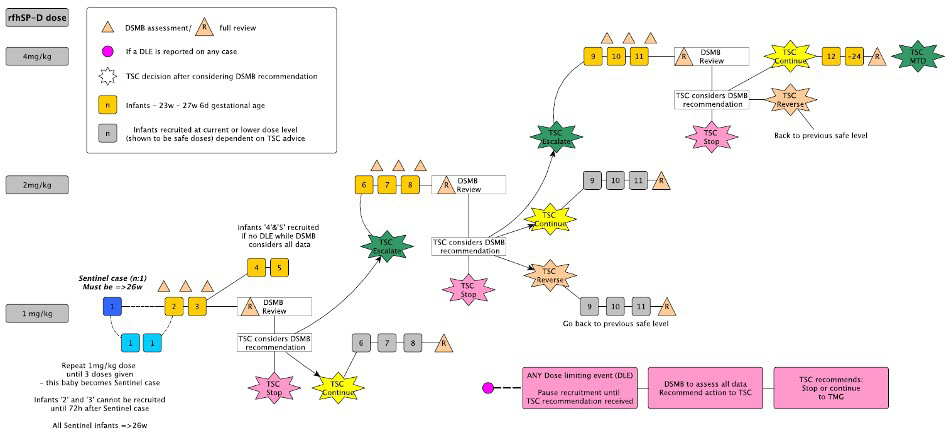
Schematic overview of the dose escalation design using the continual reassessment method. This schematic overview illustrates the decisional pathway planned. The salmon triangle refers to data review by the data and safety monitoring board (DSMB), which takes place (1) for the first three babies 72 hours after study entry after each baby at each dose level (and confirms no DLE to the management group—termed DSMB assessment), (2) after any potential DLE has been observed (and recommends trial action to trial steering committee (TSC)) and (3) in groups of three babies to make a recommendation to TSC about dose escalation. In the case of 2 or 3, a full data review by the DSMNB is undertaken (identified with a ‘R’). In the event of a suspected DLE, the DSMB will be consulted to recommend to the TSC to continue at the same dose/reduce dose and continue or trial needs to be terminated. If a DLE occurs, then the DSMB may recommend recruitment at the same dose level (eg, ‘grey’ participants) or they may advise dose de-escalation, that is, 2mg/kg down to 1 mg/kg for a further three participants before further review, after which if no DLE occurs they may advise escalation in dose again. In the absence of DLEs, the trial will continue to recruit to the same dose level during DSMB /TSC review periods.

A schema of the dose escalation procedure and review is shown in figure 2. The sentinel baby is the very first baby recruited to the study and this baby must be greater than 26 weeks GA. The sentinel baby must have received all three administrations of the IMP and have had 72 hours of observed data post administration of third administration before further infants can be recruited for the study. If the first participant does not receive all three doses of the IMP then data will still be collected but they will not qualify as the sentinel baby for this study. All infants recruited after the sentinel baby will be from 23 weeks to 29 weeks and 6 days GA for the remainder of the study. A data and safety monitoring board (DSMB) review of the Neonatal Adverse Event Severity Score (NAESS) data will take place after each infant (for the first three participants at each dose level) has received the final dose of IMP and 72 hours of monitoring. The DSMB will evaluate the safety data before further participants can be recruited, that is, the second or third baby cannot be recruited until data from the first or second baby has been reviewed. This will only be for the first three infants at each dose level, thereafter the data will be reviewed in cohorts of three unless there are safety concerns. Following the recruitment of three infants at any dose level all safety data will be reviewed by the DSMB and they will then advise the Trial Steering Committee (TSC) before a decision is made to (a) move to next dose level or (b) to stay at the same dose level or (c) decrease the dose level or (d) stop the trial. The final decision on dose escalation will be made by the TSC.

#### Continuous recruitment model during dose escalation decision period

The rationale for continuous recruitment in this trial is to minimise delays to recruitment during the DSMB review for overall dose escalation which takes place after a minimum of three participants at a dose level. It also allows for the trial of the IMP in a larger number of participants at the lower dose levels, allowing for better characterisation of the dose-response curve and the safety profile of rfhSP-D. This means that in the 1 mg/kg and 2 mg/kg cohorts, up to a further two participants can be recruited while the DSMB review dose escalation provided that no DLE has occurred in the first three participants of the dose cohort. The continued recruitment of up to two additional participants at the same or lower dose level while the DSMB conduct their review, will only be permitted if there are no concerns that a DLE has occurred in the cohort under review that is, the first 3 infants under review. Any adverse event (AE) data collected for the additional two recruited participants during DSMB review will then be reviewed by the DSMB once the 72 hours follow-up period is completed. If at any point there are concerns regarding DLEs in these additional participants, but dose escalation has occurred, then this may lead to a de-escalation. The data from the additional participants will be included at that point in the CRM which may recommend dose de-escalation in the middle of the next cohort until further data can be reviewed by the DSMB and the TSC at the next opportunity. This methodology of continual reassessment ensures that infants are only treated at the safest dose level while the safety profile is characterised.

### Study intervention and outcomes

#### Eligibility criteria

All preterm infants born before 30 weeks of gestation, intubated and treated with surfactant for RDS who are considered clinically stable are eligible. Eligibility will be confirmed within 2 hours of admission to the neonatal unit and reconfirmed for each participant before the IMP is administered.

#### Inclusion criteria

- Inborn infants born between 23 weeks and 0 days and 29 weeks and 6 days of gestation (<30 weeks), who are:
  - Intubated or intubation planned for RDS at the time of eligibility check within 12 hours from the time of birth.
  - Receiving standard surfactant replacement therapy.
  - Clinically stable on mechanical ventilation—clinical stability is defined at the time of IMP instillation and is defined below.
- Written informed consent from parents/guardians/person with legal responsibility has been given.

#### Definition of clinical stability

Eligibility of the participant must be rechecked prior to administration of the IMP given the varying clinical status of these infants. Stability will consider if the following are true:

- Blood gas parameters within the normal range for preterm infants (pH>7.20; paCO_2_<8 kPa).
- Mean blood pressure with or without inotropic support at a value in mm Hg at least numerically equivalent GA in weeks or above.
- No evidence of a pneumothorax.
- Clinical observations within acceptable range for an infant of that GA.
- The attending neonatologist considers the infant to be clinically stable.

#### Exclusion criteria

- Congenital anomalies (ie, any major antenatal diagnosed congenital abnormality) such as congenital heart disease, suspected or known chromosomal abnormalities.
- Infants requiring only non-invasive respiratory support, that is, no endotracheal intubation.
- Infants born in very poor condition and judged too sick or unstable to be included (high risk of imminent mortality) in an experimental first-in-human study; for example, infants that require maximal intensive care therapy and have findings such as a grade IV intraventricular haemorrhage that may be life-limiting.
- Infants that are born outside the participating site.
- Participation in any other interventional study (participation in another observational study is permissible).
- Parents/legal guardians are unable to give consent due to learning or other difficulties.

### Recruitment and informed consent

The study team will monitor admissions of any women in threatened or established preterm labour. Any identified women will be approached by the study team to discuss and consider the study and verbal consent for participation will be taken. All parents/legal guardians of eligible participants will be approached if the baby is born and remains eligible for informed written consent. If the person(s) providing consent on behalf of the infant does not speak English, every effort will be made to use translational service to provide an opportunity to participate in the study. If the investigator is not able to confidently take informed consent the infant will not be recruited to the study.

### Study intervention

The recombinant fragment of SP D drug product has been manufactured to good manufacturing practice and is known as rfhSP-D in this study. The IMP has orphan designation with the Food and Drug Administration. The sterile IMP is formulated in 0.9% saline at a concentration of 1 mg/mL in 2 mL vials. The first administration of rfhSP-D will be given as soon as possible after administration of standard-of-care surfactant therapy, this will be known as T0. Subsequent administration will be given at T0+12 (±2) hours and T0+24 (±2) hours. If the infant requires further standard surfactant therapy which coincides with the time of IMP administration, then the IMP should be given after the standard surfactant therapy has been administered. The IMP will be administered via a surfactant giving set that is inserted into the endotracheal tube. If the participant is extubated before any IMP dose is scheduled, then the IMP will not be administered. Vital signs will be monitored every 15 min for the first hour after administration of the IMP. The study drug can be administered by any authorised medically trained delegate. Eligibility criteria will be confirmed prior to each administration.

#### Criteria for discontinuing participation in the trial

Any dose modifications in this protocol will be in line with the trial design and according to the dose level confirmed by the DSMB and the TSC.

Reasons that the intervention may be discontinued are as follows:

- Any change in the infant’s condition that in the clinician’s opinion justifies the discontinuation of treatment.
- Withdrawal of consent for treatment by the parent/guardian/legal representative.

Participants who discontinue protocol treatment, for any of the above reasons, will remain in the trial for follow-up and data analysis. The study team does not anticipate problems with intervention adherence given that RESPONSE is an inpatient-based study. All participants in the study will receive standard neonatal care and there will be no alteration in their clinical management. The study does not require any additional follow-up of the participants recruited once they are discharged from the hospital or reach 40 weeks PMA. The hospital where the participant is being cared for is responsible for any medical care. The sponsor holds indemnity for any trial-related harm caused to the participant.

### Study outcomes

The primary outcome of this study is to assess the safety profile of rfhSP-D across three dose levels and to identify the RP2D. DLEs will be identified using clinical criteria and grading as described below.

### Dose-limiting events

The severity of all AE and/or adverse reactions (serious and non-serious) in this trial will be graded using the toxicity graded in the NAESS V.1.0.^35^ The NAESS has been developed as existing scores such as the Common Terminology Criteria for Adverse Events is not suitable for use in a study involving neonates. The NAESS developed by the International Neonatal Consortium has been developed to facilitate the conduct and appropriate interpretation of neonatal clinical trials such as RESPONSE.^36^

Grades for neonatal-specific AEs according to the NAESS V.1.0^35^ are:

Grade 1: Mild; asymptomatic or mild symptoms; clinical or diagnostic observations only; no change in baseline age-appropriate behaviours^*^; no change in baseline care or monitoring indicated.

Grade 2: Moderate; resulting in minor changes of baseline age-appropriate behaviour^*^; requiring minor changes in baseline care or monitoring^*+^.

Grade 3: Severe; resulting in major changes of baseline age-appropriate behaviour^*^ or non-life-threatening changes in basal physiological processes^+^ requiring major change in baseline care or monitoring^**_._^

Grade 4: Life-threatening; resulting in life-threatening changes in basal physiological processes^+^; requiring urgent major change in baseline care***.

Grade 5: Death.

*Age-appropriate behaviour refers to oral feeding, voluntary movements and activity, crying pattern, social interactions and perception of pain.

^+^Basal physiological processes refer to oxygenation, ventilation, tissue perfusion, metabolic stability and organ functioning.

**Minor care changes constitute: brief, local, non-invasive or symptomatic treatments.

*** Major care change constitute: surgery, the addition of long-term treatment and upscaling care level.

The DSMB will determine the occurrence of a DLE based on the following criteria:

- A single event defined as grade 3 or above on the NAESS that is possibly, probably or definitely thought to be related to the IMP. Relatedness will be confirmed by an independent neonatologist at the participating site.
- A single serious AE (SAE) that is possibly, probably or definitely thought to be related to the IMP. Relatedness will be confirmed by an independent neonatologist at the participating site.
- Concerns over frequency of any AEs reported at grade 2 on the NAESS that are possibly, probably or definitely thought to be related to the IMP.

Secondary outcomes related to efficacy for this study are as follows:

- Evaluation of systemic absorption of rfhSP-D using serial measurements of SP-D in plasma and its continued presence in tracheal fluid.
- To determine the effect of rfhSP-D on inflammatory markers in the lung secretions (eg, cell counts of neutrophils, macrophages, lymphocytes, IL-8, IL-6, IL-1).
- To compare the clinical effects of endotracheal administration of rfhSP-D on physiological and intensive care parameters in treated infants in this trial with non-treated infants from a parallel observational study.

### Participant timeline

The first administration of rfhSP-D will be given as soon as possible after administration of standard-of-care surfactant therapy, this will be known as T0. Subsequent administration will be given at T0+12 (±2) hours and T0+24 (±2) hours. Eligibility and screening investigations will be done before each administration of the IMP as shown in the schedule of events table 1.

**Table 1:** Schedule of events at screening and prior to administration of the IMP

Study visit	Screening	Baseline	Preinstillation of IMPT0	Preinstillation of IMPT0+12 hours (±2 hours)	Preinstillation of IMPT0+24 hours (±2 hours)
Informed consent	*				
Eligibility	*				
Clinical stability		*	*	*	*
Demographics (including gestational age (GA))	*	*			
Pregnancy and delivery history		*			
Stabilisation history		*			
Clinical assessment (anomalies)	*				
Vital signs	*	*		*	*
Oxygen concentration	*	*		*	*
Ventilator modality		*		*	*
Ventilator settings		*		*	*
Haematology (as per standard of care)		*			*
Biochemistry (as per standard of care)		*			*
Cytokine levels (plasma)		*			*
Cell counts GA/ETA		*		*	*
Surfactant replacement	*				
Plasma SP-D and rfhSP-D levels		*		*	*
Blood gases	*	*		*	*
SP-D levels GA		*			
rfhSP-D and SP-D level ETA				*	*
Concomitant medication		*		*	*
Cranial ultrasound scan		*			*
Review of AEs and SAEs (from time of consent)		*	*	*	*

Standard of care refers to blood samples that are taken as part of clinical care and not specific to the study.

*refers to events that must be done as per the schedule of events.

AEadverse eventETAEndotracheal AspirateGAGastric AspirateIMPinvestigational medicinal productSAEserious AESP-Dsurfactant protein D

Further clinical data will be collected as per the time points outlined in table 2 schedule of events.

**Table 2:** Subsequent time points in participant timeline following IMP administration

Study visit	T0+36 hours(±6 hours)	T0+48 hours(±4 hours)	T0+72 hours(±12 hours)	T0+96 hours(±12hours)	T0+7 days(±2 days)	T0+14 days (±2 days)T0+21 days (±2 days)T0+28 days (±2 days)	36 weeks PMA (±1 day)	40 weeks PMAor hospital discharge (±1 week)
Vital signs	*	*	*	*	*	*	*	*
Oxygen concentration	*	*	*	*	*	*	*	*
Ventilator modality	*	*	*	*	*	*	*	*
Ventilator settings	*	*	*	*	*	*	*	*
Haematology (SoC)		*	*	*	*			
Biochemistry (SoC)		*	*	*	*			
Plasma cytokine levels		*	*	*	*		*	
Plasma SP-D and rfhSP-D		*	*	*	*		*	
Blood gases		*	*	*	*	*	*	
ET cell counts	*	*	*	*	*			
ETA rfhSP-D and SP-D levels		*	*	*	*		*	
Concomitant medication	*	*	*	*	*	*	*	*
Cranial ultrasound scan		*			*	*	*	*
Walsh oxygen test							*	
Review of AEs and SAEs	*	*	*	*	*	*	*	*

*refers to events that must be done at these timepoints.

AEadverse eventETAendotracheal aspirateIMPinvestigational medicinal productSAEserious AESoCstandard of careSP-Dsurfactant protein D

The RESPONSE study will collect clinical data and biological specimens (blood, tracheal and gastric aspirates) as per tables1  2. Parents/guardians/those legally responsible for the participant will have the option to give consent for any anonymised data and samples that are collected as part of RESPONSE to be used in other ancillary studies that have ethical approval. Gastric secretions will be taken from all infants at the time of admission after placement of oro/nasogastric tube and will be discarded if consent is not obtained. Blood samples (0.5 mL of blood in an EDTA microtainer) will be collected at birth, 12 hours, 24 hours, 48 hours, 72 hours, 96 hours, day 7 and at 36 weeks PMA. Given the risk of anaemia and associated comorbidities in the study population, if there are any clinical concerns about anaemia, the clinical stability of the infant or the participant is receiving a blood transfusion the blood samples for SP-D and cytokine analysis will either be delayed or not taken.

### Data collection and management

Participants once recruited to the RESPONSE study will be allocated a study number so that all data and samples that are taken are anonymised, for example, RES_001. Participation in the clinical study will be recorded in the participant clinical records. Participants will be enrolled by the study team on the OpenClinica database. Participant clinical and laboratory data will be entered directly into the password-protected study database. After completion of the trial, the data will be exported and retained in restricted folders by the sponsor. All data will be held for 10 years following the completion of the trial.

Primary outcome data collection in this study (safety profile of rfhSP-D) will be done through grading and analysis of the incidence of DLEs. Potential causality of the DLE to the IMP will be assessed by an independent neonatologist. In addition to the DLEs, the following datasets will be collected from electronic patient records:

- At screening and on eligibility assessment: sex of participant, ethnicity, maternal medical history, antenatal steroid courses, date and time of rupture of membranes, concerns about maternal sepsis, ventilatory requirement on admission and administration of standard exogenous surfactant.

Secondary outcome data collection:

- Data will also be collected at the time points specified in the schedule of events (tables2  3) and this will include concomitant medication, ventilatory support and parameters, known positive microbiology, use of postnatal steroids, presence and treatment of pulmonary hypertension, pneumothorax, patent ductus arteriosus, use of postnatal steroids.
- At 36 weeks, PMA all participants will be assessed for severity of BPD and data will be collected about complications of prematurity such as episodes of necrotising enterocolitis and retinopathy of prematurity. The severity of BPD in participants will be defined as per the 2019 National Institute of Child health and Development (NICHD) criteria,^36^ an oxygen reduction test will be done if the participant is eligible (requiring less than Fi02<0.3 or 1.1 L/min and not on positive pressure support) and remains an inpatient at the recruiting site.

**Table 3:** Operating characteristics of the continuous reassessment model

Starting dose level 1, dose-skipping not allowed, 3 dose levels, maximum 24 participants.Skeleton (a priori probabilities of DLE) = 0.05, 0.11, 0.20target DLE rate=no greater than 20%			
	Dose level		
	1	2	3
Scenario 1: recommendation (%)	2.5	24.5	73.0
Scenario 2: recommendation (%)	22.5	46.5	31.0
Scenario 3: recommendation (%)	0.0	3.3	96.7
Scenario 1: true probabilities=a priori probabilitiesScenario 2: true probabilities=0.10, 0.20, 0.30Scenario 3: true probabilities=0.02, 0.05, 0.10			

DLEdose-limiting event

All data will be handled in accordance with the Data Protection Act 2018 and General Data Protection Regulation (GDPR) and all study members will have current GCP training and certification.

### Analysis of biological samples

Biological samples will be collected as per the schedule of events. Samples will be labelled with the participant’s study number and transported to the Targeted Lung Immunotherapy Laboratory, UCL. Surfactant components, inflammatory markers and level of SP-D will be analysed using the ELISA technique (ELLA, BioTechne) with single marker studies and multiplex assays. Cytokines to be analysed include IL-1β, IL-6, IL-8, IL-11, IL-10, IL-13, matrix metalloproteinase-9 and tumour-necrosis factor-α. Cell counts (lymphocytes, neutrophils and macrophages) in gastric and tracheal aspirates will be assessed using flow cytometry. Samples will be retained if consent has been given by the parent/legal guardian for 5 years for use in any other ethics-approved studies. If consent is not given for use in further studies or at the end of 5 years, the biological samples will be destroyed as per the laboratory standard operating procedure.

### Sample size and statistical analysis

As this is a safety study, no formal sample size calculation has been performed. A sample size of 24 infants is planned to meet practical recruitment and time targets and to collect sufficient data to quantify the estimated risk of DLE at each dose level. Participants with unclear safety outcomes or who have not started study treatment will be replaced to meet our planned effective sample size of 24 participants.

The primary outcome of interest is the occurrence of DLEs at the dose levels under investigation and the identification of the dose(s) that, for infants of particular risk profiles defined by GA, have an estimated risk of DLE closest to the target side effect level of no greater than 20%. The use of Bayesian methodology to estimate risks will allow information to be borrowed across dose levels, making the dose-escalation and RP2D identification procedure more efficient than a standard rule-based approach.

The operating characteristics of the design, for three specific scenarios, are shown in table 3. The first scenario is one where the initial a priori DLE probabilities calculated by calibration (halfwidth of the indifference interval set at 0.05) correspond to the true underlying probabilities of DLE. The second scenario is such that the true DLE rate of the second dose level corresponds to the target DLE rate. The third scenario is one where the true probabilities are much lower than the a priori probabilities.

Type and grade of DLEs, SAEs and AEs will be tabulated per dose level, and further summarised by risk group defined by GA. Mean estimated risk of DLE per dose level and 95% credibility intervals will be calculated using the study model. Secondary objectives will be described per dose level and risk category. Categorical variables will be summarised by frequencies and percentages, and continuous variables by means/medians and SD/IQRs per dose level.

#### Interim analyses of DLEs

Interim analysis will done to assess if DLEs have occurred after each infant (for the first three participants at each dose level) has received the final dose of IMP and 72 hours of monitoring and all NAESS data will be reviewed by the DSMB. The DSMB will evaluate the safety data before further participants can be recruited, this will only be for the first three infants at each dose level. The purpose of this is to ensure there are no safety concerns. For the remainder of the study, interim analysis will be done after cohorts of three participants are recruited at any dose level to assess the occurrence of DLE and review all clinical data. Overall DSMB review of all data to advise the TSC regarding dose escalation will take place after recruitment of 3 infants at any dose level. The trial statistician will calculate and provide updated summaries of the estimated risk of dose-limiting toxicity at each dose level. The DSMB will then advise if dose escalation can occur. There will be no interim analysis for the secondary outcomes.

The study will be terminated if any of the following stopping rules are satisfied:

- There is at least a 90% chance that the risk of DLE at dose level 1 is greater than the target of 20%. If the trial is terminated under this rule, no drug dose will be recommended due to safety concerns.
- The number of participants who have been treated without side effects is deemed sufficient.
- There is evidence of increased mortality or morbidity in the participants treated with the IMP.

### Study oversight and monitoring

The sponsor will provide trial oversight and verify the trial processes and prompting corrective action to the clinical study team as required. An independent TSC will provide oversight of the trial to safeguard the interests of the trial participants. The TSC will also provide advice to the chief investigator, CCTU and the funder on all aspects of the trial through its independent chair. An independent DSMB is assigned with an allocated chair. The DSMB will be responsible for monitoring and accumulating the safety data and making recommendations to the TSC on whether the trial should continue as planned. The DSMB will consider data as per the statistical analysis and make recommendations to the TSC chair for consideration by the TSC.

### Patient and public involvement

The TSC has a patient representative and the patient-facing documents have been reviewed and commented on by the patient representatives.

#### AE reporting

All AEs grade 3 or above on the NAESS/SAE will be reported to CCTU within 24 hours until the participant reaches day 7 following the last administration of the IMP (preclinical studies have demonstrated that the IMP is not detectable in plasma sample taken 24 hours after final administration). All related events that are graded 1 or 2 according to the NAESS will be reported within 7 days. After day 7 any events that are considered related to the IMP will be reported within 24 hours of knowledge to the sponsor. Assessments for and reporting of all AEs related/unrelated will continue until 40 weeks PMA or hospital discharge. All aggregated AEs data will be considered by the DSMB at each meeting to confirm that there are no trends, safety signals or safety concerns. Examples of AEs that are exempt from reporting are those that are graded 1 or graded 2 according to the NAESS criteria if considered not related to the IMP. These are common observations in preterm infants and do not require a change in clinical management unless sustained, that is, grade 3 and above on the NAESS. There is no formal frequency of audit for this study but will be overseen by the sponsor and if required by the Medicine and Healthcare products Regulatory Agency.

### Significance of study

Despite the medical advances in neonatal medicine, the incidence of BPD has not changed over the years, and one may argue that it has increased because we see an increasing number of extremely preterm infants survive to discharge. The lifelong morbidity associated with BPD has significant implications for healthcare systems around the world.

Infants at the highest risk of BPD are born at a GA when the majority of the alveolar and vascular development in the lungs occurs. Immaturity of the lungs means they have a developmental deficiency of surfactant leading to RDS. Ongoing lung injury secondary to postnatal insults such as infection and mechanical ventilation leads to ongoing interruption to lung development. Efforts have been made over the years to reduce these insults by changes in ventilation strategies, early nutrition and proactive management of infection in the hope that the lungs will repair and remodelling will lead to recovery of the lung parenchyma. However, a significant number of preterm infants will have multiple insults and despite best efforts will have abnormal repair with little lung recovery leading to BPD. Inflammation remains at the centre of the pathophysiology of BPD and the most promising target for therapies. Given this, there is a need for novel anti-inflammatory therapies to be explored such as SP-D.

The role of SP-D in lung immune homeostasis is well established but due to its propensity to oligomerise does not lend itself well to a stable drug form. The proposed recombinant fragment of SP-D has been developed into a stable drug form for endotracheal administration and animal studies in a well-established translational model have demonstrated its potential anti-inflammatory effects. This phase I safety study using dose escalation of 1–4 mg/kg will aim to identify a recommended phase 2 dose for a subsequent randomised phase 2 study in preterm infants born at less than 28 weeks gestation who are at the highest risk of developing neonatal chronic lung disease.

## Ethics and dissemination

All results and analyses from the study will be published in peer-reviewed journals and presented at national and international conferences. All data generated in this study will be anonymised and the study will be conducted per Good Clinical practice. Access to the full study protocol will be given on request and participant-level data will only be given if authorised by the sponsor for auditing purposes. Any substantial or non-substantial amendment to the study must be approved by the Health Research Authority and will be communicated with the NHS trust research and development team to ensure site implementation. Any study material that is related to participant information or informed consent will be submitted to the principal research ethics committee for approval. This study has been approved by London-Brent NHS Research Health Authority ethics committee (REC reference 23/LO/0381) and has clinical trials approval (CTA 20363/0453/001-0001) in place.
