# Supplementary material for: Recombinant fragment of human surfactant protein D to prevent neonatal chronic lung disease (RESPONSE): a protocol for a phase I safety trial in a tertiary neonatal unit
Source: BMJ Open. 2024 Aug 16;14(8):e086394. doi: 10.1136/bmjopen-2024-086394 (PMC11331837; doi:10.1136/bmjopen-2024-086394)
Supplement: Supplementary file 1 [file bmjopen-2024-086394.reviewer_comments.pdf]

PEER REVIEW HISTORY

BMJ Open publishes all reviews undertaken for accepted manuscripts. Reviewers are asked to complete a checklist review form (**http://bmjopen.bmj.com/site/about/resources/checklist.pdf**) and are provided with free text boxes to elaborate on their assessment. These free text comments are reproduced below.

ARTICLE DETAILS

**TITLE (PROVISIONAL)**	Recombinant Surfactant protein D to prevent neonatal chronic lung disease (RESPONSE): a protocol for a phase I safety trial in a tertiary neonatal unit.
**AUTHORS**	Bhatt, Reena; Madsen, Jens; Castillo-Hernandez, Tania; Chant, Kathy; Dehbi, Hakim-Moulay; Marlow, Neil; Clark, Howard

VERSION 1 – REVIEW

**REVIEWER**	Hillman, Noah H<br>Saint Louis University
**REVIEW RETURNED**	10-Apr-2024

**GENERAL COMMENTS**	The three treatments (T0, T12, T24) of the drug per dose (labelled doses) gets a little confusing for the three different doses (1 mg/kg, 2 mg/kg, 4 mg/kg) since both are labeled “three doses” in the diagrams.<br><br>Figure 2 is a little confusing. The light grey star object and dark grey start object are both labelled TSC Continuous, even when the DLE event started. Especially at patients 11-13, where patients are on both of the 2 mg/kg and 4 mg/kg dose without an eventual connect to the higher doses. This might need some clarification<br><br>The patient data was easy to follow. Great job on it.<br><br>There were no expected dates of the trials in the protocol.

**REVIEWER**	Perrone, Serafina<br>University of Parma Department of Medicine and Surgery
**REVIEW RETURNED**	21-Apr-2024

**GENERAL COMMENTS**	The rationale for the study is sound, and the paper potentially addresses important research questions.<br>The paper is well written<br>However, I have some concerns about this manuscript, primarily regarding methodology<br>In this study, a recombinant fragment of surfactant protein D drug product will be used. Has this product been registered anywhere?<br>Additionally, how much blood is needed for all the measurements? Four blood draws are scheduled in the first week of life, a period in which extremely low birth weight infants are prone to developing anemia and requiring blood transfusions. Blood transfusions and oxidative stress are two main factors critically involved in neonatal bronchopulmonary dysplasia.<br>Pag 10: secondary objective are explained.<br>Serial measurements of SP-D in plasma and in endotracheal

	secretions<br>Inflammatory markers in lung secretion<br>In the text, it has been stated that cytokine levels will be measured in plasma; please clarify.<br>How long will the infants be followed up?<br>How will all these variables be utilized?<br>Minor:<br>There are some abbreviations that are unnecessary because they are mentioned only once in the text, such as MHRA and HRA

VERSION 1 – AUTHOR RESPONSE

Reviewer: 1
Dr. Noah H Hillman, Saint Louis University
Comments to the Author:
The three treatments (T0, T12, T24) of the drug per dose (labelled doses) gets a little confusing for the three different doses (1 mg/kg, 2 mg/kg, 4 mg/kg) since both are labeled “three doses” in the diagrams.
Thank you for your comments, we have amended the figures and hope that this is now better explained for the reader.

Figure 2 is a little confusing. The light grey star object and dark grey start object are both labelled TSC Continuous, even when the DLE event started. Especially at patients 11-13, where patients are on both of the 2 mg/kg and 4 mg/kg dose without an eventual connect to the higher doses. This might need some clarification
Thank you for your comments, we have amended the figure to reflect a change in the frequency of the drug safety monitoring board reviews. We hope that this makes the schematic for the study clearer.

The patient data was easy to follow. Great job on it.
Thank you.

There were no expected dates of the trials in the protocol.
We have added in the methods section the date the study was opened and the planned recruitment period.

Reviewer: 2
Dr. Serafina Perrone, University of Parma Department of Medicine and Surgery
Comments to the Author:
The rationale for the study is sound, and the paper potentially addresses important research questions.
The paper is well written
Thank you for your comments. We have addressed each of the concerns that you have presented below.

In this study, a recombinant fragment of surfactant protein D drug product will be used. Has this product been registered anywhere?
The recombinant fragment of surfactant protein D drug product is not registered as it is currently being tested for safety in this phase I safety trial. The drug has orphan designation with the FDA. We have amended the text to include that it has orphan designation (under the section titled study intervention).

Additionally, how much blood is needed for all the measurements? Four blood draws are scheduled in

the first week of life, a period in which extremely low birth weight infants are prone to developing anaemia and requiring blood transfusions. Blood transfusions and oxidative stress are two main factors critically involved in neonatal bronchopulmonary dysplasia.
Thank you for your comment. The amount of blood required is 0.5mls and is taken when other blood tests are required. If there are concerns that the baby is anaemic and extra samples will be detrimental, the sample is not taken. When consenting the parents/carers/guardians are shown the sample table and are aware we will only take the sample if the baby is stable and at the time of any other blood draws required for monitoring such as a blood gas. We have amended the text to reflect the volume of blood required under the section ‘participant timeline’.

Pag 10: secondary objective are explained.
Serial measurements of SP-D in plasma and in endotracheal secretions
Inflammatory markers in lung secretion
In the text, it has been stated that cytokine levels will be measured in plasma; please clarify.
Thank you. We have amended the secondary objective to reflect that the cytokines will be measured in the lung secretions and plasma.

How long will the infants be followed up?
All infants will be followed up until discharge from the hospital or they reach 40 weeks postmenstrual age. We have amended the text to reflect this under the section ‘Criteria for discontinuing participation in the trial’.

How will all these variables be utilized?
All variables that are collected are used to assess whether an adverse event has occurred and also to fulfil the secondary objective which is to compare the clinical effects of endotracheal administration of rfhSP-D on physiological and intensive care parameters in treated infants in this trial with non-treated infants from a parallel observational study.

Minor:
There are some abbreviations that are unnecessary because they are mentioned only once in the text, such as MHRA and HRA
We have removed these from the abbreviations.

We hope that our responses above and the changes to the original manuscript are acceptable. We would once again like to thank you for your time and consideration of this manuscript and very much hope that it will be accepted for publication. We have attached a marked copy and a clean copy of the revised manuscript below.

Yours sincerely,
Dr Reena Bhatt on behalf of all authors.

VERSION 2 – REVIEW

**REVIEWER**	Hillman, Noah H<br>Saint Louis University
**REVIEW RETURNED**	28-May-2024

**GENERAL COMMENTS**	The authors have addressed my concerns and the concerns of the other reviewer.
